# The prognostic impact of previously infectious complications on allogeneic hematopoietic stem cell transplantation for patients with severe aplastic anemia: A single-center, retrospective study

**DOI:** 10.3389/fimmu.2022.1004787

**Published:** 2022-09-12

**Authors:** Yuanfeng Zhang, Xin Chen, Donglin Yang, Aiming Pang, Rongli Zhang, Qiaoling Ma, Weihua Zhai, Yi He, Jialin Wei, Erlie Jiang, Mingzhe Han, Sizhou Feng

**Affiliations:** ^1^ State Key Laboratory of Experimental Hematology, National Clinical Research Center for Blood Diseases, Haihe Laboratory of Cell Ecosystem, Institute of Hematology and Blood Diseases Hospital, Chinese Academy of Medical Sciences and Peking Union Medical College, Tianjin, China; ^2^ Department of Hematology, The Affiliated Yantai Yuhuangding Hospital of Qingdao University, Yantai, China

**Keywords:** aplastic anemia, infection, stem cell transplantation, comparison, survival

## Abstract

Whether infections before transplantation impair the survival of patients with severe aplastic anemia (SAA) remains unclear. The aim of this retrospective cohort analysis was to compare survival between patients with SAA who underwent allogeneic hematopoietic stem cell transplantation (allo-HSCT) with infection (n=66) and patients without infection (n=189) from one medical center. There were no differences in baseline characteristics, except that more patients in the infection group were diagnosed with VSAA (59.09% *vs*. 30.69%, *P*<0.001), and their grafts were more peripheral blood stem cells (89.39% *vs*. 76.72%, *P*=0.042). In addition, the percentage of patients with multidrug-resistant organism colonization or infection in the infection group was larger (16.7% *vs*. 0.5%, *P*<0.001). The median days of engraftment were similar between the two groups; however, the 28-day engraftment rates of neutrophils and platelets were lower in the infection group. No differences were observed in terms of grades II–IV acute graft-versus-host disease (aGVHD) (*P*=0.418), grades III–IV aGVHD (*P*=0.075), mild to severe chronic GVHD (cGVHD) (*P*=0.899), and moderate to severe cGVHD (*P*=0.342). Patients in the infection group had more bloodstream infections before engraftment (28.8% *vs*. 15.3%, *P*=0.016), and the primary cause of death was infection instead of aGVHD in contrast to patients without infection (16.7% *vs*. 4.2%, *P*=0.002). Finally, the estimated overall survival (OS), failure-free survival (FFS), and GVHD-free FFS at 5 years were 63% (95% CI, 51–78), 60% (95% CI, 47–74), and 55% (95% CI, 43–70) in patients with infection before transplantation versus 86% (95% CI, 81–92) (*P*<0.001), 82% (95% CI, 76–88) (*P*<0.001), and 75% (95% CI, 69–82) (*P*=0.003) in patients without infection before transplantation, respectively. Multivariate analysis identified haploidentical HSCT and pre-HSCT anti-infection response, defined as partial remission (PR) or stable disease (SD), as adverse factors of OS and FFS. In conclusion, our study demonstrated that SAA patients with infection defined as PR or SD but not complete remission before allo-HSCT showed inferior survival compared with patients without infection. Therefore, more attention should be paid to prophylaxis and complete control of infectious complications before transplantation among SAA patients.

## Introduction

Severe aplastic anemia (SAA) is a life-threatening disease caused mainly by fetal bleeding and recurrent infections due to persistent severe cytopenia. The cornerstone of treatment is considered to reserve normal hematopoiesis by modulating the immune environment through intensive immunosuppressive therapy (IST) or regenerating new blood cells by allogeneic hematopoietic stem cell transplantation (allo-HSCT). Although IST is equal to allo-HSCT in terms of overall survival (OS), its disadvantage is significantly lower failure-free survival (FFS), and treatment failures include no hematological response, relapse, and clonal evolution (1–4); however, the drawbacks of allo-HSCT are high transplant-related mortality (TRM) and potential long-term complications. Patients with previous infections may also have an influence on their choice of therapy. Theoretically, immune and blood count recovery are fundamental treatments for infections. Indeed, compared with IST, allo-HSCT has the advantage of rapid blood recovery, which may outbalance the risk of TRM. Taking advances from supportive care to optimization of conditioning, graft-versus-host disease (GVHD) prophylaxis, and alternative donor transplantation, especially haploidentical donor HSCT (HID-HSCT), SAA patients with infections may be considered a candidate for allo-HSCT. Here, we designed this single-center retrospective study to assess whether infections before allo-HSCT impact the survival of patients with SAA undergoing allo-HSCT.

## Patients and methods

### Patients

We collected the clinical data of patients diagnosed as acquired AA and consecutively receiving allo-HSCT from the Institute of Hematology and Blood Diseases Hospital, Chinese Academy of Medical Sciences and Peking Union Medical College from 2005 to 2020. Of the 255 patients enrolled, 189 patients have not experienced infection before transplantation, while 66 patients suffered infection previously. The latter were considered as those experiencing any infection from diagnosis to conditioning. Inclusion criteria included patients diagnosed as SAA, very SAA (VSAA), or transfusion dependence non-SAA (NSAA), and absence of pregnancy. Exclusion criteria included inherited bone marrow failure syndrome and severe organ dysfunction. Owing to time cost in searching a matched unrelated donor (MUD) and great advancement in HID-HSCT in China, few patients received MUD-HSCT during study period and were not enrolled into this research. All patients or their relatives signed the written informed consent. This study was approved by the Ethics Committees of the Institute of Hematology and Blood Diseases Hospital, Chinese Academy of Medical Sciences and Peking Union Medical College.

### Prophylaxis for fungal infection and treatment of infection before allo-HSCT

Since the diagnosis of SAA, prophylaxis for fungal infection was not the routine in our center except when IST was administered. When SAA patients have fever, empirical broad-spectrum antibiotics according to the guideline was given (5, 6). Then, modification guided by clinical response, radiographic findings, and microbiologic cultures was performed.

### Transplantation

In our center, an SAA patient of age ≤50 years with a donor would receive MSD-HSCT as the first-line therapy, while older patients or patients without an MSD could undergo MSD-HSCT or HSCT from alternative donors as a salvage therapy after IST failure, and sometimes as the first-line therapy according to the intension of the patients.

Conditioning regimens, GVHD prophylaxis, and infection prophylaxis were consistent with our previous studies (4, 7).

Since 2019, we have performed rectal and nasal swabs to screen multidrug-resistant organism (MDRO) colonization before transplantation and then routinely screened for MDRO every week.

### Definitions

A temperature of ≥38.3°C for ≥1 h was considered to be fever (8).The definitions of SAA, VSAA, and NSAA were in accordance with the guideline (9). The duration of administrating antibiotics or antifungal against infection was considered as the course of anti-infection. Acute GVHD (aGVHD) and chronic GVHD (cGVHD) were defined according to guidelines (10, 11). The definitions of invasive fungal diseases (IFDs) were in accordance with a consensus, which consisted of proven, probable, and possible IFD (12). Anti-infection responses were classified as complete remission (CR), partial remission (PR), or stable disease (SD) according to the literature (13). CR was defined as the disappearance of all clinical, microbiological, and radiological criteria; PR was defined as an improvement in the above criteria; and SD was defined as no improvement in the criteria above. Cytomegalovirus (CMV) viremia was monitored by plasma CMV DNA testing with real-time PCR every week; it was considered positive when >600 copies/mL CMV in two consecutive tests or >1000 copies/mL CMV in a single test was detected on peripheral blood as previously reported (14), and preemptive therapy was initiated in high-risk patients. The days of neutrophil and platelet engraftment were defined as the first day of three consecutive days with neutrophil count >0.5×10^9^/L unsupported by G-CSF and seven consecutive days with platelet count >20×10^9^/L without transfusion. Primary graft rejection (GR) was defined as failure of neutrophil engraftment until day 28, whereas secondary GR was defined as loss of graft function after initial engraftment. TRM was defined as death without disease progression. OS was defined as survival at the last follow-up. Post-transplantation treatment failures include death, GR, relapse, or clone evolution. FFS was defined as survival with a response. GVHD-free and failure-free survival (GFFS) was defined as survival with response and without grades III–IV aGVHD or moderate to severe cGVHD.

### Statistical analysis

The main objective of this study was to compare the outcomes of patients with SAA with or without infections before allo-HSCT.

The patients included had follow-up through electronic databases, outpatient departments, or telephones. The final follow-up was conducted on February 31, 2022. Groups were compared using the Mann–Whitney U test for continuous variables and the chi-square or Fisher’s exact test when the number of subjects was fewer than 5 for categorical variables. The median follow-up was calculated using the reverse Kaplan–Meier method. Gray’s test was used to calculate the cumulative incidence of GVHD, with death from any cause and GR as competing events. The Kaplan–Meier method was used to estimate the probabilities of OS and FFS, and the groups were compared using the log-rank test. Variables with *P* values ≤ 0.05 in the univariate analysis were included in a Cox proportional hazards model to identify factors impacting OS and FFS. Data analysis was performed using R software (R 4.1.2), SPSS 20.0, and GraphPad Prism 5. Figures were generated using GraphPad Prism 5. All *P* values were two-sided, and factors were recognized as independent predictors when the *P* value was <0.05.

## Results

### Baseline characteristics

As shown in **Table 1**, no differences were observed between the two groups in the donor type, patient age, patient sex, donor sex, presence of paroxysmal nocturnal hemoglobinuria clones, previous IST, interval from diagnosis to transplantation, donor age, anti-thymocyte globulin source, conditioning regimen, years of transplantation, and amounts of mononuclear cells and CD34^+^ cells infused. However, more patients in the infection group were diagnosed with VSAA (59.09% *vs*. 30.69%, *P*<0.001) and received peripheral blood stem cells as grafts (89.39% *vs*. 76.72%, *P*=0.042). In addition, the percentage of patients with MDRO colonization or infection in the infection group was larger (16.7% *vs*. 0.5%, *P*<0.001). The details of the patients with previous infections are shown in **Table 2**. Before transplantation, 18, 42, and 6 patients had CR, PR, and SD, respectively, according to the anti-infection response. Invasive pulmonary fungal diseases, bacterial pneumonia, and bloodstream infections (BSIs) account for the majority of infections. Among the 51 evaluable patients, 45 were administered with carbapenem antibiotics, and the median course of anti-infection was 54 days, ranging from 3 to 324 days. One patient with VSAA presented with invasive pulmonary and paranasal sinus fungal diseases and soft tissue and gingival infection at diagnosis, and his anti-infection course persisted for nearly 1 year before allo-HSCT. At the last follow-up 769 days post-HSCT, the patient was alive without active infection.

**Table 1:** Characteristics and outcomes of patients with acquired aplastic anemia.

Variables	Without infections (n = 189)	With infections (n = 66)	*P* value
Donor type, no. (%)			0.54
MSD	119 (62.96)	45 (68.18)	
HID	70 (37.04)	21 (31.82)	
Patient age, years, median (range)	22.5 (3.5–53.5)	26.5 (3.8–54.7)	0.054
Patient gender (man), no. (%)	108 (57.14)	45 (68.18)	0.153
Donor gender (man), no. (%)	96 (50.79)	34 (51.52)	1
Diagnosis, no. (%)			< 0.001
Severe aplastic anemia	114 (60.32)	25 (37.88)	
Very severe aplastic anemia	58 (30.69)	39 (59.09)	
Non-severe aplastic anemia	17 (8.99)	2 (3.03)	
Presence of PNH clones, no. (%)	29 (15.34)	9 (13.64)	0.893
Previous IST, no. (%)	11 (5.82)	8 (12.12)	0.16
MDRO colonization or infection	1 (0.5)	11 (16.7)	< 0.001
Interval from diagnosis to transplant, months, median (range)	3 (0.8–247)	3 (1–231.2)	0.95
Blood types of donors to recipients, no. (%)			0.65
Matched	110 (58.20)	40 (60.61)	
Major mismatched	33 (17.46)	13 (19.70)	
Minor mismatched	32 (16.93)	7 (10.61)	
Major and minor mismatched	14 (7.41)	6 (9.09)	
Donor age, years, median (range)	28.3 (7–55.3)	30.6 (8.6–62.4)	0.126
ATG source, no. (%)			0.439
pALG	85 (44.97)	34 (51.52)	
rATG	104 (55.03)	32 (48.48)	
Conditioning regimen, no. (%)			1
ATG+CTX+FLU	133 (70.37)	46 (69.70)	
BU+FLU+ATG+CTX	56 (29.63)	20 (30.30)	
Graft source, no. (%)			0.042
Peripheral blood	145 (76.72)	59 (89.39)	
Bone marrow ± peripheral blood	44 (23.28)	7 (10.61)	
Mononuclear cells infused, ×10^8^/kg, median (range)	8 (3.1–25.5)	8.5 (2.8–23.1)	0.423
Year of transplantation			0.626
2005–2015	86 (45.50)	33 (50.00)	
2016–2020	103 (54.50)	33 (50.00)	
CD34^+^ cells infused, ×10^6^/kg, median (range)	2.9 (1.5–8.6)	2.9 (1.6–5.9)	0.724
Blood-stream infection before engraftment	29 (15.3)	19 (28.8)	0.016
Early deaths before engraftment	0 (0)	6 (9.09)	< 0.001
Neutrophil engraftment, days, median (range)	12 (8–23)	12 (9–23)	0.888
Platelet engraftment, days, median (range)	13 (7–83)	15 (9–201)	0.164
28-day neutrophil engraftment, no. (%)	188 (99.47)	61 (92.42)	0.005
28-day platelet engraftment, no. (%)	162 (85.71)	48 (72.73)	0.028
Graft rejection, no. (%)			0.89
Primary graft rejection	2 (1.06)	1 (1.67)	
Secondary graft rejection	8 (4.23)	2 (3.33)	
Cytomegalovirus viremia, no. (%)	79 (41.80)	18 (27.27)	0.052
100-day grades II–IV aGVHD, no. (%) ^*^	38 (20.54)	15 (25.86)	0.5
100-day grades III–IV aGVHD, no. (%) ^*^	14 (7.61)	9 (15.52)	0.125
Mild-to-severe cGVHD, no. (%) ^#^	26 (15.03)	8 (17.02)	0.914
Moderate-to-severe aGVHD, no. (%) ^#^	8 (4.62)	4 (8.89)	0.453
Overall deaths, no. (%)	25 (13.23)	22 (33.33)	< 0.001
Follow-up of alive patients, months, median (range)	37 (4–169)	37.4 (4.5–123)	0.735

*Among the enrolled patients, 184 and 59 patients were evaluable; ^#^among the enrolled patients, 173 and 48 patients were evaluable. no., number of patients; MSD, matched sibling donor; HID, haploidentical donor; PNH, paroxysmal nocturnal hemoglobinuria; pALG, porcine antilymphocyte globulin; rATG, rabbit antithymocyte globulin; CTX, cyclophosphamide; FLU, fludarabine; BU, busulfan; aGVHD, acute graft-versus-host disease; cGVHD, chronic graft-versus-host disease.

**Table 2:** Clinical information of patients with infections.

Variables	No. (%)
Anti-infection responses, no. (%)	
Complete remission	18 (27.3)
Partial remission	42 (63.6)
Stable disease	6 (9.1)
Site of infection, no. (%)	
Bloodstream	22 (33.3)
Bacterial pneumonia	23 (34.8)
Pulmonary IFD (proven)	4 (6.1)
Pulmonary IFD (probable)	15 (22.7)
Pulmonary IFD (possible)	7 (10.6)
Gingiva and tonsil	13 (19.7)
Skin and soft tissue	7 (10.6)
Perianal	4 (6.1)
FUO	3 (4.5)
Appendix and urinary tract	2 (3.0)
Liver and spleen	2 (3.0)
≥3 sites involved, no. (%) ^*^	12 (23.5)
Carbapenem antibiotics administered, no. (%) ^*^	45 (88.2)
MDRO infection or colonization, no. (%) ^*^	11 (21.6)
Course of anti-infection, days, median (range), no. (%) ^*^	54 (3–324)

*51 patients were evaluated. IFD, invasive fungal disease; FUO, fever of unknown origin disease; MDRO, multidrug-resistant organisms.

### Hematopoietic recovery

No differences were observed between the two groups with regard to the GR. One and two patients experienced primary GR, while two and eight patients experienced secondary GR in the infection and non-infection groups, respectively (*P*=0.89). On day 28 post-transplantation, neutrophil and platelet engraftment was achieved in 61 (92.42%) and 48 (72.73%) of 68 patients in the infection group versus 188 (99.47%) (*P*=0.005) and 162 (85.71%) (*P*=0.028) of 188 patients in the non-infection group. The median time for neutrophil and platelet recovery was 12 (range, 9–23) and 15 (range, 9–201) days in the infection group compared with 12 (range, 8–23) and 13 (range, 7–83) days in the non-infection group, respectively, which showed no statistically significant difference.

### Infections

More patients in the infection group were subjected to BSIs before engraftment (28.8% *vs*. 15.3%, *P*=0.016), while there was no statistical difference between the two groups with regard to CMV viremia post-transplantation (41.8% *vs*. 27.27%, *P*=0.052).

### GVHD

**Figure 1:**
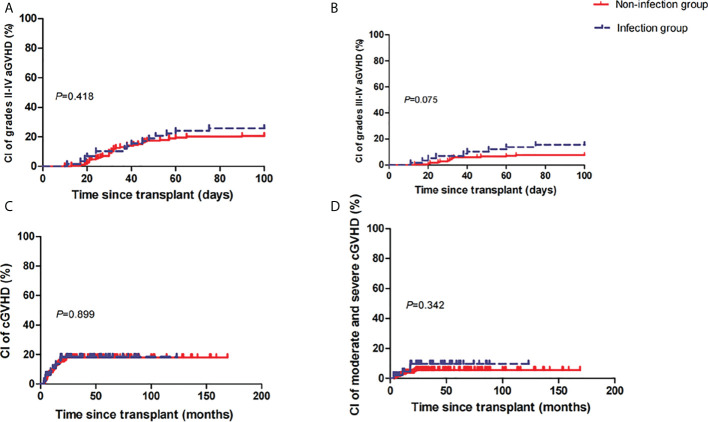
Cumulative incidences (CIs) of grades II–IV acute graft-versus-host disease (aGVHD) **(A)**, grades III–IV aGVHD **(B)**, mild-to-severe chronic GVHD (cGVHD) **(C)**, and moderate-to-severe cGVHD **(D)** between the two groups.

At 100 days post-HSCT, the cumulative incidences of grades II–IV and grades III–IV aGVHD were 26% (95% confidence interval [CI], 7–41) and 15% (95% CI, 0–28) in the infection group versus 21% (95% CI, 10–30) (*P*=0.418) (**Figure 1A**) and 8% (95% CI, 0–14) (*P*=0.075) (**Figure 1B**) in the non-infection group. At 5 years post-HSCT, the cumulative incidences of mild-to-severe cGVHD and moderate-to-severe cGVHD were 18% (95% CI, 0–33) and 10% (95% CI, 0–19) in the infection group versus 18% (95% CI, 5–28) (*P*=0.899) (**Figure 1C**) and 6% (95% CI, 0–11) (*P*=0.342) (**Figure 1D**) in the non-infection group, respectively.

### Deaths

In total, 22 and 25 patients died in the infection and non-infection groups, respectively, and the leading primary causes of death in the two groups were infection (n=11) and aGVHD (n=11), respectively (**Table 3**). More patients in the infection group died due to infection (16.7% *vs*. 4.2%, *P*=0.002). Strikingly, there were six early deaths before engraftment in the infection group compared with none of the patients in the non-infection group (*P*<0.001, **Table 1** **)**. Among the 11 patients with MDRO colonization or infection before transplantation in the infection group, 4 patients developed BSIs caused by the same pathogen. Eventually, six patients died: infection in four patients, aGVHD in one patient, and GR in one patient.

**Table 3:** Primary causes of death (COD) among patients.

COD	Patients with previous infections (n = 22) (%)	Patients without previous infections (n = 25) (%)	*P* value
Infection	11 (16.7)	8 (4.2)	0.002
Bloodstream	5	–	
Pulmonary	5	5	
Maxillofacial	1	–	
EBV	–	2	
Cranial	–	1	
aGVHD	6 (9.1)	11 (5.8)	0.392
cGVHD	1 (1.5)	3 (1.6)	1
Accident	1 (1.5)	1 (0.5)	0.451
Intracranial hemorrhage	1 (1.5)	1 (0.5)	0.451
Graft failure	2 (3)	1 (0.5)	0.165

EBV, Epstein–Barr virus; aGVHD, acute graft-versus-host disease; cGVHD, chronic graft-versus-host disease.

### Survival

**Figure 2:**
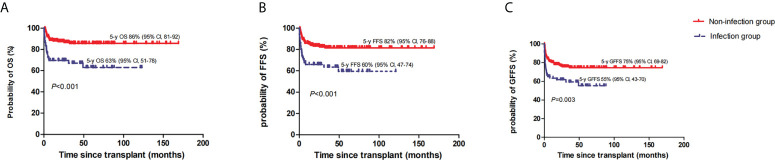
The estimated 5-year overall survival (OS) **(A)**, failure-free survival (FFS) **(B)**, and GVHD-free, failure-free survival (GFFS) **(C)** between the two groups.

The estimated OS rates at 5 years in the infection group were 63% (95% CI, 51–78) and 86% (95% CI, 81–92) in the non-infection group (*P*<0.001) (**Figure 2A**). Accordingly, the estimated FFS and GFFS rates at 5 years were 60% (95% CI, 47–74) and 55% (95% CI, 43–70), and 82% (95% CI, 76–88) (*P*<0.001) (**Figure 2B**) and 75% (95% CI, 69–82) (*P*=0.003) (**Figure 2C**), respectively. Moreover, the estimated OS rate at 5 years among VSAA patients with infection (n=39) was 56% (95% CI, 36–76), which was lower than SAA or NSAA patients with infection (n=27) (74%, [95% CI, 61–94]); however, the *P* value was not significant (0.332). In multivariate analysis, HID-HSCT and anti-infection response, defined as PR or SD, respectively, were adverse factors of OS and FFS (**Table 4**).

**Table 4:** Univariate and multivariate analysis of survival.

Variables	Comparison	Overall survival				Failure-free survival			
		Univariate analysis		Multivariate analysis		Univariate analysis		Multivariate analysis	
		HR (95%CI)	*P* value	HR (95%CI)	*P* value	HR (95%CI)	*P* value	HR (95%CI)	*P* value
Donor source	HID *vs* MSD	2.23 (1.25–3.96)	0.006	2.32 (1.24–4.34)	0.009	2.21 (1.3–3.74)	0.003	2.68 (1.51–4.77)	<0.001
Patient sex	Woman *vs* man	0.6 (0.32–1.13)	0.112	–	–	0.61 (0.34–1.07)	0.087	–	–
Donor sex	Woman *vs* man	0.9 (0.51–1.59)	0.716	–	–	0.81 (0.48–1.36)	0.421	–	–
Patient age	Continuous variable	1.04 (1.01–1.06)	0.003	1.02 (1–1.05)	0.051	1.02 (1–1.04)	0.034	1.01 (0.99–1.04)	0.265
Presence of PNH	Yes *vs*. No	1.3 (0.61–2.79)	0.501	–	–	1.05 (0.5–2.23)	0.897	–	–
Diagnosis (VSAA)	VSAA *vs* SAA	1.47 (0.81–2.65)	0.204	–	–	1.62 (0.94–2.79)	0.082	–	–
Diagnosis (NSAA)	NSAA *vs* SAA	1.08 (0.32–3.6)	0.906	–	–	1.31 (0.45–3.76)	0.619	–	–
Treatment	ATG *vs* no ATG	1.26 (0.45–3.5)	0.664	–	–	1 (0.36–2.76)	0.998	–	–
ATG source	rATG *vs* pALG	0.97 (0.54–1.71)	0.906	–	–	1.16 (0.68–1.97)	0.582	–	–
Anti-infection response	CR *vs* without	2.05 (0.71–5.9)	0.182	1.89 (0.62–5.78)	0.263	1.55 (0.55–4.38)	0.411	1.15 (0.38–3.47)	0.8
Anti-infection response	PR or SD *vs* without	3.25 (1.77–5.97)	<0.001	2.99 (1.47–6.09)	0.003	2.9 (1.66–5.07)	<0.001	2.99 (1.57–5.72)	<0.001
MDRO colonization or infection	Yes *vs* No or unknown	4.79 (2.02–11.33)	<0.001	1.6 (0.6–4.27)	0.352	4.15 (1.77–9.71)	0.001	1.94 (0.77–4.91)	0.16
Donor age	Continuous variable	1.03 (1.01–1.06)	0.011	1 (0.97–1.03)	0.963	1.03 (1–1.05)	0.017	1.01 (0.98–1.03)	0.642
Interval from D to T	Continuous variable	1 (1–1.01)	0.137	–	–	1 (1–1.01)	0.24	–	–
Year of transplantation	2015–2020 *vs* 2005–2015	2.29 (1.23–4.26)	0.009	1.71 (0.86–3.37)	0.124	1.62 (0.94–2.78)	0.081	–	–
Graft source	BM ± PB *vs* PB	0.54 (0.23–1.26)	0.154	–	–	0.6 (0.28–1.27)	0.184	–	**-**
Amount of MNC	Continuous variable	1.06 (0.99–1.13)	0.089	–	–	1.03 (0.97–1.1)	0.314	–	–
Amount of CD34^+^ cells	Continuous variable	1.2 (0.96–1.5)	0.108	–	–	1.15 (0.93–1.42)	0.203	–	–

HR, hazard ratio; CI, confidence interval; HID, haploidentical donor; MSD, matched-sibling donor; PNH, paroxysmal nocturnal hemoglobinuria; VSAA, very severe aplastic anemia; SAA, severe aplastic anemia; NSAA, non-severe aplastic anemia; ATG, antithymocyte globulin; rATG, rabbit ATG; pALG, porcine antilymphocyte globulin; CR, complete remission; PR, partial remission; SD, stable disease; D, diagnosis; T, transplantation; BM, bone marrow; PB, peripheral blood; MNC, mononuclear cells.

## Discussion and conclusions

Whether and when to perform allo-HSCT for patients with SAA with infection remains uncertain. Sometimes, it is difficult to control infections in an immunocompromised state, and recurrent infection is an intractable issue. In contrast to IST, allo-HSCT can rapidly restore hematopoiesis. Nevertheless, we suggest that patients with SAA and incompletely controlled infections before transplantation experienced inferior survival.

In our study, we noticed that patients with VSAA were more likely to have infections before transplantation. This finding is in line with the results of Liu et al. (15). In addition, the absolute neutrophil count may represent the potential residual hematopoiesis of bone marrow, and VSAA patients may exhibit a poor response to IST (16, 17). Therefore, prompt infection prophylaxis and timely effective therapy for patients with VSAA may reduce the risk of infection and improve survival. Alternative donors HSCT, including HID-HSCT, can be considered for patients without an MSD (2, 3, 18, 19). It has the advantages of universal and immediate availability of donors. In our previous study, we showed that HID-HSCT had a comparable OS but superior FFS compared with IST (4). Xu et al. (2) and Liu et al. (3) also reported similar results, demonstrating that HID-HSCT was superior to IST in terms of FFS. However, in the current study, we identified HID-HSCT as a risk factor for survival, which was different from that reported by Xu et al. (18). This difference may be ascribed to HID-HSCT as salvage therapy and a longer interval from diagnosis to therapy in our patients (5 months *vs*. 1.5 months) (4).

In contrast to other studies (15, 20), we found that infection, defined as PR or SD, was an adverse factor of OS and FFS. Xu et al. showed that 51 SAA patients with PR or SD before transplantation had similar results in terms of 3-year OS (85.4% *vs*. 92.9%, *P*=0.530) and 3-year FFS (82.7% *vs*. 92.9%, *P*=0.458) compared with 14 patients with CR before transplantation. MDRO information was not available in this study, and the only factor impacting OS in the multivariate analysis was an ECOG score of 3 pre-HSCT (20). Liu et al. also reported comparable survival of 130 SAA patients with grade I to IV infections, defined by the Common Terminology Criteria for Adverse Events Version 4.0, versus 300 SAA patients without infection. They also found that more patients died from infection in the infection group (14.29% *vs*. 5.33%, *P*=0.002), as demonstrated in our study (15). The reason for these variances may be that more patients had severe and complex infections in our study. A total of 11 patients had MDRO colonization or infection, and 12 patients had greater than or equal to three sites of infection. In addition, our patients with infection had a longer interval from diagnosis to transplantation (median of 3 months). From another point of view, more patients in our study encountered pre-engraftment BSIs than in the study by Liu et al (28.8% *vs*. 20.3%) (15).

There were several reasons for the higher mortality rate in the infection group. First, more attention should be paid to patients with MDRO colonization or infection. As reported, among acute myeloid leukemia patients undergoing allo-HSCT, the 5-year OS was inferior for those with MDRO colonization compared with non-colonized patients (43.3 *vs*. 65.5%, *P*=0.002), mainly due to higher non-relapse mortality (33.9 *vs*. 9.4%, *P*<0.001) related to infections (15.5% *vs*. 4.9%) (21). Girmenia et al. (22) also reported that colonization with gram-negative MDRO is significantly associated with an increased incidence in BSIs caused by the same pathogen. Studies have also demonstrated that broad-spectrum antibiotic exposure is a risk factor for MDRO colonization, leading to MDRO infections (23–25). In our research, we noticed that 4 out of 11 patients with MDRO colonization or infection developed BSIs caused by the same pathogen, and 3 patients died. Second, patients in the infection group had more pre-engraftment BSIs, which is related to a higher mortality rate (26). Moreover, Girmenia et al. revealed that pre-engraftment gram-negative BSIs were an adverse factor related to death 4 months after allo-HSCT, and gram-negative BSIs accounted for most of our positive cases (15 out of 19 patients). Third, a higher proportion of patients in the infection group experienced grades III–IV aGVHD, but without significant differences between the two groups (*P*=0.075) and deaths due to aGVHD (*P*=0.392). Loss of intestinal diversity caused by broad-spectrum antibiotic exposure has been associated with increased aGVHD (27, 28).

Our study had several limitations. First, as this was a retrospective study, selection bias was unavoidable. The higher number of peripheral blood stem cell recipients in the infection group may have led to a higher cumulative incidence of GVHD. Even so, we did not observe statistical difference of grades III–IV aGVHD between the two groups. Second, the advent of drugs for fungal prophylaxis and treatment has contributed to the reduced mortality associated with fungal infections in the past decade. In our study, four patients died before 2010 due to invasive pulmonary (n=3) or cerebral fungal diseases (n=1). However, we could not perform further analyses because of the small sample size.

In conclusion, our study demonstrated that SAA patients with infection defined as PR or SD but not CR before allo-HSCT showed inferior survival compared with patients without infection. Therefore, more attention should be paid to prophylaxis and complete control of infectious complications before transplantation. However, there are no defined recommendations for the prophylactic antibiotic or antimycotic drugs in SAA patients after diagnosis (9, 29). In the future, prospective studies to access the optimal strategy for infection prophylaxis of SAA patients should be explored.

## Data availability statement

The raw data supporting the conclusions of this article will be made available by the authors, without undue reservation.

## Ethics statement

This study was reviewed and approved by Ethics Committees of Institute of Hematology and Blood Diseases Hospital, Chinese Academy of Medical Sciences and Peking Union Medical College. Written informed consent to participate in this study was provided by the participants’ legal guardian/next of kin.

## Author contributions

SF contributed to the study design and manuscript review. YZ and XC contributed to the data collection and analysis. YZ wrote the manuscript and performed the statistical analyses. XC, DY, AP, RZ, QM, WZ, YH, JW, EJ, MH, and SF contributed to the disease treatment and data collection. All authors contributed to the manuscript and approved the submitted version.

## Funding

This work was supported by the Haihe Laboratory of Cell Ecosystem Innovation Fund (grant number HH22KYZX0036) and the CAMS Innovation Fund for Medical Sciences (CIFMS) (grant numbers 2021-I2M-1-017 and 2021-I2M-C&T-B-080).

## Acknowledgments

We are grateful to the patients and their family members for their trust in our study.

## Conflict of interest

The authors declare that the research was conducted in the absence of any commercial or financial relationships that could be construed as a potential conflict of interest.

## Publisher’s note

All claims expressed in this article are solely those of the authors and do not necessarily represent those of their affiliated organizations, or those of the publisher, the editors and the reviewers. Any product that may be evaluated in this article, or claim that may be made by its manufacturer, is not guaranteed or endorsed by the publisher.
